# Characterisation of Mixing in the Proximal Duodenum of the Rat during Longitudinal Contractions and Comparison with a Fluid Mechanical Model Based on Spatiotemporal Motility Data

**DOI:** 10.1371/journal.pone.0095000

**Published:** 2014-04-18

**Authors:** Clément de Loubens, Roger G. Lentle, Corrin Hulls, Patrick W. M. Janssen, Richard J. Love, J. Paul Chambers

**Affiliations:** 1 INRA, AgroParisTech, UMR 782 Génie et Microbiologie des Procédés Alimentaires, 78850, Thiverval-Grignon, France; 2 Institute of Food, Nutrition and Human Health, Massey University, Palmerston North, New Zealand; 3 Institute of Veterinary, Animal and Biomedical Sciences, Massey University, Palmerston North, New Zealand; University of Zurich, Switzerland

## Abstract

The understanding of mixing and mass transfers of nutrients and drugs in the small intestine is of prime importance in creating formulations that manipulate absorption and digestibility. We characterised mixing using a dye tracer methodology during spontaneous longitudinal contractions, i.e. pendular activity, in 10 cm segments of living proximal duodenum of the rat maintained ex-vivo. The residence time distribution (RTD) of the tracer was equivalent to that generated by a small number (8) of continuous stirred tank reactors in series. Fluid mechanical modelling, that was based on real sequences of longitudinal contractions, predicted that dispersion should occur mainly in the periphery of the lumen. Comparison with the experimental RTD showed that centriluminal dispersion was accurately simulated whilst peripheral dispersion was underestimated. The results therefore highlighted the potential importance of micro-phenomena such as microfolding of the intestinal mucosa in peripheral mixing. We conclude that macro-scale modeling of intestinal flow is useful in simulating centriluminal mixing, whereas multi-scales strategies must be developed to accurately model mixing and mass transfers at the periphery of the lumen.

## Introduction

Modelling the transit and digestion of material in the small intestine by different theoretical chemical reactors or compartmental models [1] has been useful in the development of drugs [2] and foods [3]–[5] enabling their absorption and digestibility to be characterised [6]. The output of these compartmental models depends upon the flow of material between each reactor, the chemical reactions, the absorption kinetics and the intensity of mixing by molecular diffusion and/or contractile activity within each reactor. These theoretical models can be refined and parameterised by input from separate *in vivo* or *in vitro* experiments that focus on the contribution of specific mechanisms [7]. They are also useful in assessing the deviation of real systems from the theory [8].

Fluid mechanical analysis of flow within particular compartments of the gastro-intestinal system [1], [9]–[17] has allowed the influence of their patterns of contractile activity on the mixing of the lumen content to be explored. Modelling of flow during axial propulsion from concerted propagating circular and longitudinal contractions [12], [18], [19], i.e. peristaltic activity, has shown the presence of vortices that redistribute the lumen content [11], [12]. The results of spatio-temporal (ST) analysis of non propagating longitudinal contractions [20], i.e. pendular activity, in the duodenum of the rat [10] was recently incorporated in simulations using lattice Boltzmann numerical methods [21]. The results from this study indicated that pendular activity could generate longitudinal dispersion of the luminal content by shear deformation in absence of propulsion [10]. However these findings were not validated experimentally.

One method that can be used for experimental validation of such simulations is based on chemical reactor engineering principles [8], and involves determining the amounts of time that ‘marked’ elements of fluids spend in an isolated intestinal segment [9]. The ‘Residence Time Distribution’ (RTD) of elements of fluid within the lumen varies with the degree of axial and radial dispersion generated by particular flow conditions and with molecular diffusivity. The use of this method in conjunction with ST mapping of motility [22] has shown that, in the terminal ileum of the possum, viscous fluids are less prone to mixing by convection during peristalsis than are fluids of lower viscosity [9].

In a prior analysis of pendular activity in the duodenum of the rat [10], flow and mixing characteristics were postulated to reflect the process of longitudinal array of continuously stirred tank reactors (CSTRs) with ongoing slow axial transfers of reactants between them. The current study was undertaken to test this hypothesis. Firstly mixing during pendular activity in the proximal duodenum of the rat was characterised by chemical reactor engineering principles. Secondly, the theoretical reactor configuration that most closely fitted the resulting RTD dye elution curve was identified [8]. Thirdly we incorporated real time motility data of the proximal duodenum into a computationnal fluid dynamical (CFD) model (19,20) to obtain ‘CFD RTD profiles’ and compared them with the experimental curves.

## Methods

### Preparation of duodenal segments

All the experimental procedures were approved by the Massey University Animal Ethics Committee (MUAEC approval n° 12/45), and complied with the New Zealand Code of Practice for the Care and Use of Animals for Scientific Purposes.

Seven Sprague Dawley rats were each anaesthetised with halothane and dissected following the method of our earlier work [23], [24]. The peritoneal cavity was opened with a vertical incision. A 10 cm length of gut that extended from a point just distal to the pyloric sphincter was resected along with its peritoneal attachment, flushed clean of digesta and cannulated at both the oral and the aboral ends before being installed in a 300 mL organ bath containing HBS solution [23], [24] maintained at 37°C, which was continuously oxygenated (95% O_2_, 5% CO_2_) and recirculated at a flow rate of 160 mL/min. The animal was subsequently euthanised with intracardiac pentobarbitone (125 mg/kg).

### Experimental Residence Time Distribution (RTD)

We restricted our analysis to fluids of low viscosity on the grounds that significant quantities of liquids are ingested and secreted into the stomach [25] and the proximal duodenum [26], [27] during the post-prandial period (i.e. digestive phase after ingestion of a meal rich in nutrients). Furthermore our previous modelling study had shown that an increase in the viscosity of the lumen content had limited effects on mixing [10].

**Figure 1:**
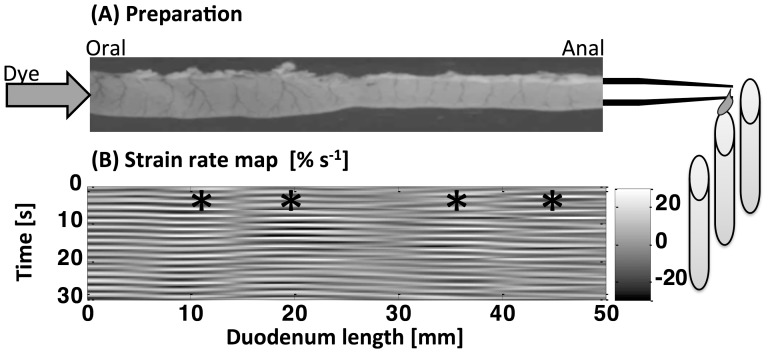
Schema showing the ex vivo preparation and the system for perfusion and collection of the effluent (A) and spatiotemporal L map of non propagating longitudinal contractions, i.e. pendular activity, in the proximal duodenum of the rat (B). (A) The duodenal segment is immersed in, and superperfused with, carboxygenated Earles Hepes solution maintained at 37°. Effluent is collected every minute from the distal conical cannula tip.(B) Darker shades indicate areas of relaxation (positive longitudinal strain rate, in s^−1^) and lighter shades areas of contraction (negative longitudinal strain rate). The L map presents four spatial domains (*) in which the longitudinal strain rate oscillates between positive and negative values.

The perfusion assembly was designed to deliver the tracer fluid directly into the proximal end of the duodenum in a manner that avoided any mixing of the dye step interface (see below) prior to or during entry. The distance between the ends of the proximal and distal cannulae was adjusted to prevent the duodenal segment from bending laterally and sagging by more than 2 cm [20]. A conical outflow pipe was mounted on the outlet of the distal cannula to allow the effluent perfusate to be collected drop by drop into collection tubes (Fig. 1).

The duodenal segment was perfused with clear saline at flow rates of 0.16 mL/min for 15 minutes to allow recovery of the organ from the effects of anaesthesia which was generally characterized by the resumption of regular longitudinal contractions. The flow rate was chosen to mimic that of pyloric outflow during the post-prandial period in the rat [10], [28]. The syringe containing the perfusate was then switched to one containing normal saline coloured with 1.4 mg/mL Methylene Blue (BDH Laboratory Supplies, Poole, UK) to create a step increase in its concentration. The effluent was collected every 60 s in order to have a sufficient volume for its analysis. The dye concentration in each sample was determined in a U-2001 spectrophotometer (Hitachi, Ibaraki, Japan) at 630 nm following dilution of 30 µL of sample with 1470 µL of normal saline.

The same procedures were repeated to determine the step response RTD of a length of silicone tubing of similar length (10 cm) and internal diameter (3 mm) to that of the segments of the duodenum. This was done to assess the degree of axial dispersion in the experimental set-up.

### Analysis of step response RTD

**Figure 2:**
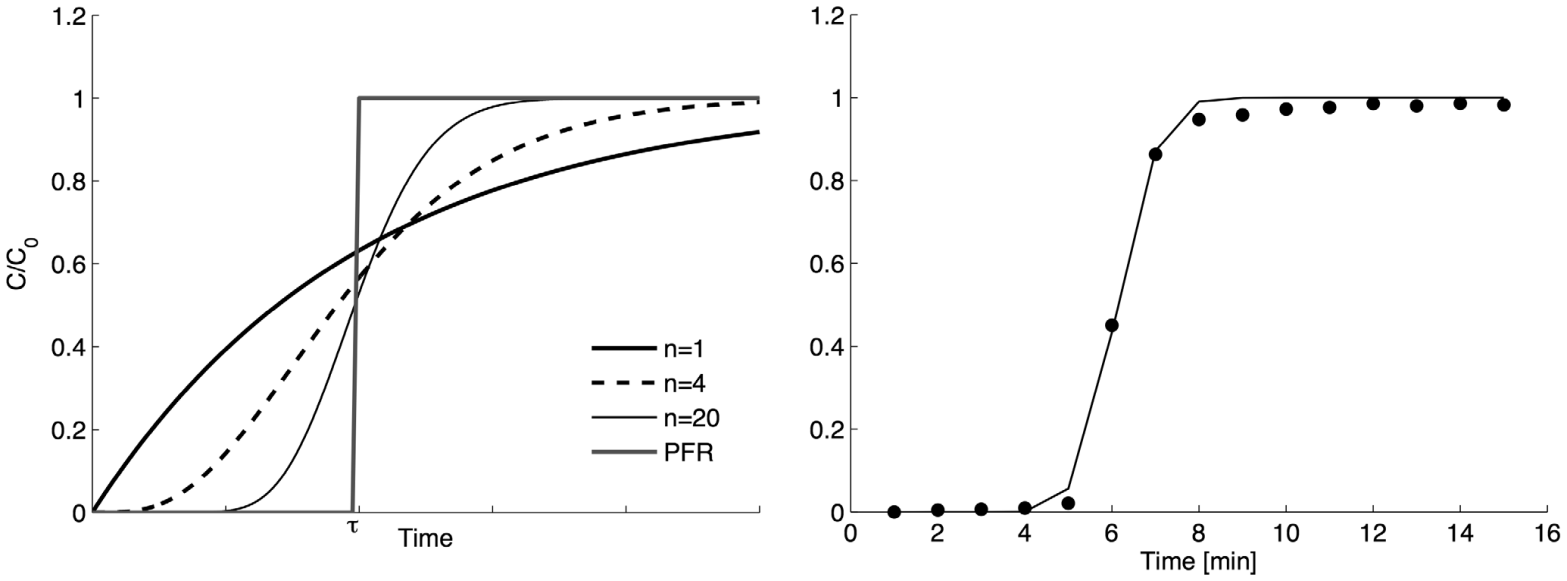
Theoretical step response RTDs and determination of minimal axial dispersion in the experimental layout. Left: Theoretical step response RTD for different numbers (n) of continuous stirred tanks reactors (CSTRs) in series. Plug flow reactor (PFR) configuration (no axial but perfect radial mixing) is approached as the number of CSTRs tends to infinity. Right: Experimental step response RTD (plain circles) after passage through a length of silicone rubber tubing of similar dimensions and flow rate to those of the isolated proximal duodenum. The line of best fit was for 68 CSTRs in series (R^2^ = 0.99, plain line).



In a single CSTR, mixing by advection and/or diffusion is instantaneous across its dimensions (Fig. 2). As the number of CSTRs in series increases along a given length of intestine, the intensity of the axial dispersion will decrease (Fig. 2). As the number of CSTRs tends to infinity the configuration will approach that of a Plug Flow Reactor (PFR) with perfect radial mixing and no axial dispersion. The general form of the function predicting RTD step responses is

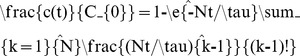
(Eq.1)where c is the concentration of the effluent, C_0_ the concentration of the probe, t is the time (in s), N is the number of CSTRs and τ is a time constant given by the ratio of the total volume of the reactors divided by the flow rate. In order to mimic the mixing resulting from the collection process occurring every minute, a moving average filter with a time step of 1 min was then applied to (Eq. 1).

The experimental F curves that were obtained for each animal, were averaged for each time step to determine the optimal parameters of the model (i.e. N and τ). Optimal values of τ were firstly determined by fitting (Eq. 1) to the averaged experimental data for a value of N between 1 and 100. Secondly, the couple (N,τ) that gave the greatest value of R^2^ allowed us to identify the optimal values of CSTR models.

### Image acquisition and processing

Full sequences of images of the section of the duodenal segment were acquired. A video camera (Basler scA1000-20fc, Ahrensburg, Germany) with a zoom lens (Cosmicar 12.5–75 mm) was mounted 450 mm above the organ bath which captured monochrome images at a rate of 5 frames per second and wrote these to hard disk as uncompressed TIF format files. This procedure yielded the high quality images necessary for generating high-fidelity maps with one pixel corresponding to 0.13 mm. Diameter (D map) and longitudinal strain rate (L map) maps were derived as described in earlier work [23], [24]. Briefly, D maps describe variations in the diameter of duodenum, i.e. constrictions and dilatations at all points along its length over time. L maps describe the rate of local lengthening or shortening (i.e. strain rate) at all points along the long axis of the duodenum over time. The quality of the images allowed small differences in the vascular arcades on the duodenal wall to be resolved. Thus, it was possible to determine relative longitudinal movement by cross-correlation between successive frames based on movements of particular vascular patterns. The longitudinal strain ε was defined by ΔL/L where L is the length of a segment of muscle and ΔL is its change of length due to muscle contraction. The longitudinal strain rate dε/dt (in s^−1^) is defined as the time derivative of the strain ε, i.e. the rate of local lengthening (if dε/dt<0) or shortening (if dε/dt>0). The D maps from each experimental run were screened to ensure that no peristaltic contractions occurred during the period in which the step response RTD was determined. Similarly all L maps were screened to ensure that ongoing stationary fast phasic longitudinal contractions [20] persisted throughout the experimental procedure (Fig. 1).

### Modelling of fluid mechanical consequences of pendular contractile activity in the duodenal lumen

A fluid mechanical model of small intestinal flow previous published by our group [10] was adapted to predict F curves, thereafter termed ‘CFD RTD’. The model used lattice-Boltzmann (LB) numerical methods [21] to simulate the flow and the convection of a diffusive tracer in the duodenal lumen. The velocity boundary conditions at the walls were determined by using real sequences of longitudinal contractile activity. Briefly, the fluid was assumed to be Newtonian and the geometry of the lumen was modelled as a tube of 4 mm diameter. The Navier-Stokes equations were solved on a 2 dimensional Cartesian mesh by the LB methods for incompressible flow [29] and the longitudinal axis was assumed to be an axis of symmetry. The moving boundary conditions imposed by the longitudinal contractions and the oral and aboral pressures and velocity conditions were modelled by the methods of Zou and He [30]. The advection-diffusion equations were solved by one particular member of the family of 2D advection-diffusion LB algorithms described by Ginzburg [31] called ‘optimal advection.’ Five sequences of L maps of 15 minutes duration were used to simulate the dispersion of a diffusive tracer that was perfused at a constant flow rate.

Oral and aboral boundary conditions that reproduced the experimental conditions were applied. Hence the velocity profile at the inlet was assumed to be parabolic (i.e. Poiseuille flow) and the concentration of the tracer was equal to 1. Null radial velocity components, zero pressure and zero-diffusive flux boundaries were imposed at the aboral end. The fluid viscosity was set to 1 mPa.s (i.e. viscosity of water) and the diffusion coefficient of methylene blue in water was set to 5×10^−10^ m^2^/s (calculated from the Stokes-Einstein formula, [32]). The concentration of the dye in the outflow as a function of the time was then calculated and a moving average filter with a time step of 1 min was applied to the resulting RTD curves in order to mimic the collection process every minute.

## Results and Discussion

### Experimental step response RTD

The step response RTD determined on flow through a silicon rubber tube (Fig. 2) was best modeled by a high number (68 tanks) of CSTRs (R^2^ = 0.99). This indicates that the flow through the system approached that of a PFR and that the experimental set-up generated minimal inherent axial dispersion.

**Figure 3:**
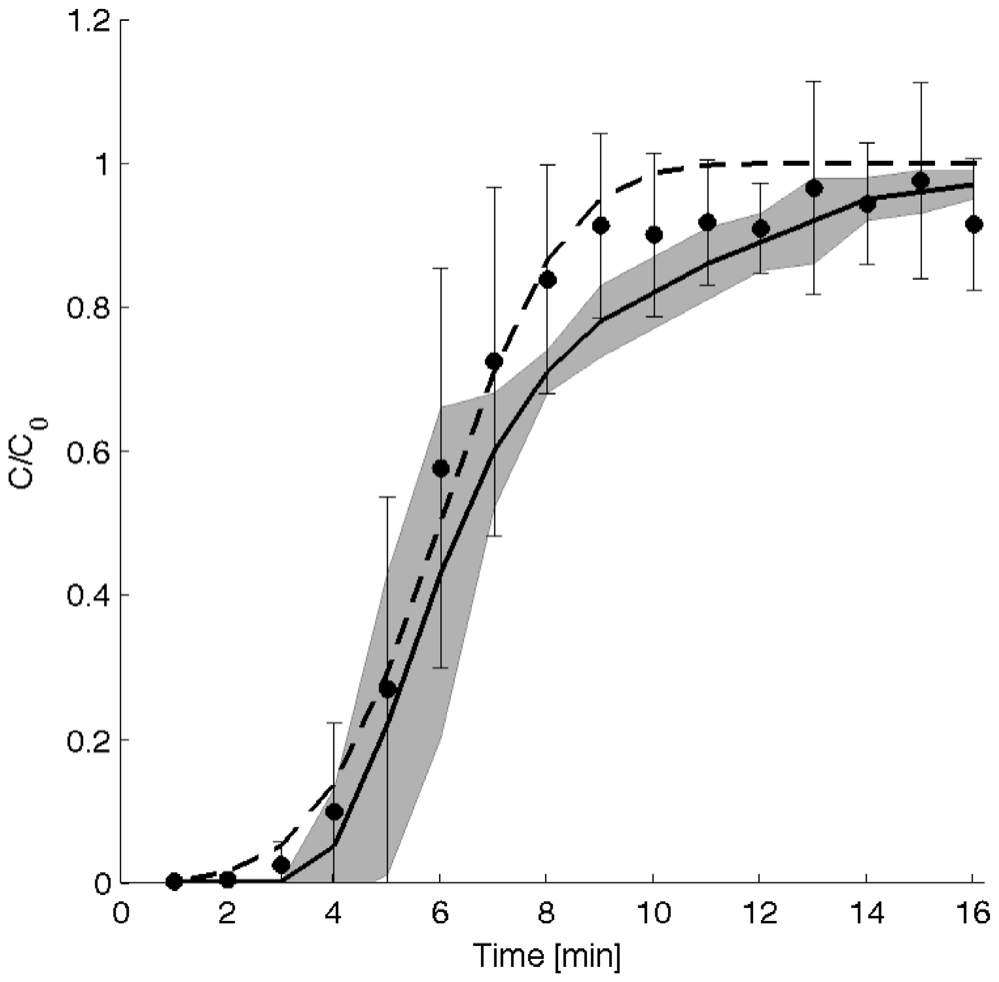
Comparisons of experimental RTD values from the isolated proximal duodenum of the rat with theoretical RTD and the response predicted by the computational fluid dynamical (CFD) model. The experimental RTD (plain circles, error bar  =  +/−s.d.) curve was best fit by the theoretical RTD curve (dashed line) of output from 8 continuously stirred tank reactors in series (R^2^ = 0.98, 7 rats). Sequences of real longitudinal contractile activity were incorporated into the CFD model simulating the RTD responses after stepped dye input at time zero (plain line: mean value for 5 rats, limits of the shaded envelope +/− s.d.). The experimental data (plain circles, error bars: +/− s.d.) lay within the s.d. for time periods below 7 min, whereas they lay outside the s.d. of the simulations at times between 7 and 11 minutes after step delivery. This discrepancy could be attributed to the absence of mechanisms such as microfolds in the model, that could increase mixing at the wall.

Ongoing non-propagating longitudinal activity occurred with no peristalsis during the time of measurement in seven *ex vivo* preparations of the duodenum (Fig. 1). The number (3–5 domains) and amplitude (0.3–0.5 s^−1^) of the spatial domains of longitudinal activity varied with the time and between preparations as detailed in our previous work [20]. There was greater axial dispersion of the probe in the duodenal preparations than in the silicon tube as was indicated by the sigmoidal shape of the RTD curve (Fig. 3). RTD curve was best fitted by 8 CSTRs in series (R^2^ = 0.98, n  =  7 rats) (Fig. 3). The value of C/C_o_ on exit remained always below 1, contrary to that of the silicon rubber tube. Hence this was likely to result from absorption of the tracer by the mucus layer [33] on the wall of the intestine.

A plug flow reactor system (PFR) has been postulated to be the most ‘efficient’ configuration for digestion in the small intestine as incoming substrate is mixed radially with no axial dispersion so that the length of the reactor required to completely admix enzymes with incoming substrate and absorb nutrient products is minimised [6]. The fact that the experimentally determined RTD (F curve) were best fitted by the calculated output from a small number (8 tanks) of CSTRs in series, indicates that mixing in the proximal duodenum does not approach that generated by a PFR as was suggested by previous workers [1], [6]. It is noteworthy the number of CSTRS did not correspond with the number of longitudinal contractile domains along the preparation (3–5 domains). Hence it seems unlikely that each contractile domain functioned simply as a CSTR. Given the spontaneous, presumably stochastic, variation of the ST charactersitics of the contractile activity [10], [34], it seems more likely that mixing resulted from the overall effect of the ensemble in randomly generating regions of shear.

### Comparison with CFD modelling

The F curves obtained from the contractile model were also sigmoidal in shape (Fig. 3). At times below 7 minutes, the F curves of the contractile models were in good agreement with those of the experimental data. After 7–11 minutes of tracer delivery, the experimental results tended to exceed the F curves simulated by the contractile model.

**Figure 4:**
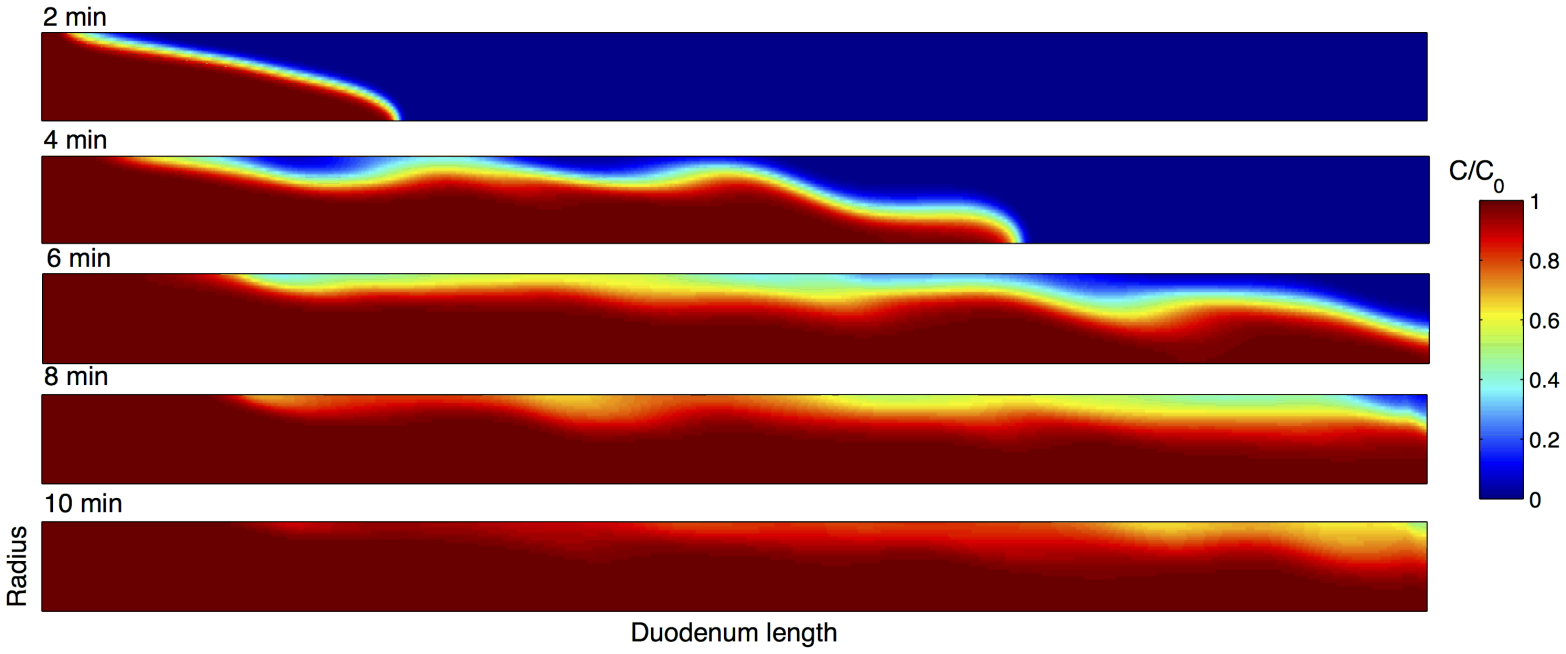
Results from fluid mechanical simulation of the dispersion of the probe in the duodenum of the rat during pendular activity using the CFD model showing the pattern of dispersal 2, 4, 6, 8 and 10 min after the delivery of the step in dye input injection. Pendular activity increased the dispersion in the periphery more than in the center of the lumen. The top boundary is the intestinal wall; the bottom boundary is central luminal axis and the direction of flow is from left to right. The horizontal axis is assumed to be an axis of symmetry.

Isovalues of tracer concentration in the lumen (Fig. 4) showed that the dispersion was augmented by pendular activity, principally in the periphery of the lumen. Comparison with the experimental RTD showed that centriluminal dispersion was accurately simulated by the macro-scale CFD model whilst peripheral dispersion was underestimated.

Recent findings have suggested that differences in mechanical properties of the mucosal, submucosal and smooth muscle layers of the small intestine could augment peripheral mixing by generating temporary microfolding of the mucosa when the muscular layer is contracting [35]. Thus folding of the mucosal layers causes villi to crowd together alternately expelling and pumping fluid from the interstitial spaces [35]. The presence of discrete viscoelastic regions of mucins suspended in a continuous fluid of Newtonian viscosity around the mucosa [36] may also augment peripheral mixing. Currently the inclusion of such micro phenomena in a macroscopic model of the small intestine flow and mixing is challenging due to a finite limitation in computational resources. Strategies that employ multi-scale modeling [16], [37] would be required.

Together these results show that the use of chemical engineering methods to quantify the outcome of mixing within a closed system by the use of dye tracers in *ex vivo* preparations of the small intestine is well suited to the testing of the veracity of CFD models. Hence these preparations allow the delivery of the dye marker to be closely controlled in situations that are generally analogous to the conditions in the model in that the effects of secretion and absorption of significant quantities of fluid are obviated.

## Conclusion

In conclusion, the use of dye tracer techniques shows that the outcome of mixing in the proximal duodenum during continued pendular contractile activity does not approach that of a PFR configuration and is equivalent to that of a low number of CSTRs. Further, that whilst models based on the pattern of pendular contractile activity in the duodenum are adequate to model centriluminal flow, they tend to underestimated peripheral mixing. This may be due to the effects of micro phenomena as microfolding [35] and/or gel filtration [36] which are not considered in these models.
