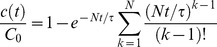# Correction: Characterisation of Mixing in the Proximal Duodenum of the Rat during Longitudinal Contractions and Comparison with a Fluid Mechanical Model Based on Spatiotemporal Motility Data

**DOI:** 10.1371/journal.pone.0105239

**Published:** 2014-08-07

**Authors:** The PLOS ONE Staff

There is an error in Equation 1. Please see the correct Equation 1 here.